# Deep learning model for diagnosing lupus erythematosus in cardiac patients using ECG and audio spectrograms

**DOI:** 10.1038/s41598-025-14128-3

**Published:** 2025-09-29

**Authors:** Atef F. Hashem, Abdirashid M. Yousuf, Ahmed Hassan

**Affiliations:** 1 Department of Mathematics and Statistics, College of Science, Imam Mohammad Ibn Saud Islamic University (IMSIU), Riyadh, 11432 Saudi Arabia; 2 Research and Innovation Center, Amoud University, Amoud Valley, Borama, 25263 Somalia; 3 Faculty of Science, Beni-Suef University, Beni-Suef, 62511 Egypt

**Keywords:** Electro diagrams, Deep learning, Explainable deep learning, Heart patients, Biomedical engineering, Diagnosis, Health services, Medical imaging

## Abstract

Individuals with both Lupus Erythematosus and pre-existing heart conditions are more likely to develop severe symptoms, emphasizing the complex and not fully understood interaction between the disease and cardiovascular health. A universal diagnostic model based on fixed rules has proven ineffective, as demonstrated in the experimental section of this study. To address this challenge, we propose an efficient and novel approach. Our model consists of two complementary subsystems. The first leverages Residual Network (ResNet) to capture complex patterns within ECG datasets, capitalizing on its ability to identify complex patterns in sequential data. The captured features are subsequently processed through Long Short-Term Memory (LSTM) networks. The second subsystem takes an alternative approach, we introduce a novel pipeline that converts ECG images into audio, enabling Mel-spectrogram generation and deep analysis via a fine-tuned Audio Spectrogram Transformer (AST). This audio-based representation reveals richer temporal and spectral features, leading to more accurate and interpretable classification than traditional methods. Experimental findings indicate that our hybrid approach achieves exceptional performance, with accuracy, sensitivity, specificity, and AUC scores of 99%, 99.2%, 96.8%, and 97%, respectively. Furthermore, we validate our model’s effectiveness through an explainable deep learning framework using a heatmap algorithm. The results suggest that Lupus Erythematosus may contribute to ventricular hypertrophy, as indicated by the model’s emphasis on the QRS region in ECG images from the test dataset.

## Introduction

Deep learning has significantly transformed the healthcare sector, offering advanced solutions for complex medical challenges. Deep learning models have consistently outperformed traditional approaches in comparison to human medical experts in specific diagnostic tasks. This advantage extends beyond speed—an essential factor in medical decision-making—to exceptional accuracy. Additionally, deep learning models provide continuous availability, unlike human doctors, and consistently achieve higher accuracy rates ^22^.

Lupus Erythematosus (LE) is a chronic autoimmune disorder in which the body’s immune system mistakenly attacks healthy tissues and organs, with Systemic Lupus Erythematosus (SLE) being its most prevalent and severe form. This disease can affect multiple systems, including the skin, joints, kidneys, brain, and heart, often presenting with a wide range of symptoms that complicate its diagnosis. Traditionally, the diagnosis of SLE involves a combination of clinical assessment and laboratory testing. Physicians evaluate symptoms such as a butterfly-shaped rash on the face, joint pain, fatigue, fever, photosensitivity, and potential signs of kidney involvement. Laboratory tests play a central role in confirming the diagnosis, with antinuclear antibodies (ANA) serving as a common initial marker, though not specific to lupus. More specific antibodies such as anti-double-stranded DNA (anti-dsDNA) and anti-Smith (Sm) are used alongside complement protein levels (C3, C4), inflammatory markers like ESR and CRP, and urinalysis to detect kidney damage. In certain cases, imaging studies and tissue biopsies, especially of the kidneys, may be performed. To aid diagnosis, clinicians often rely on established classification systems such as the American College of Rheumatology (ACR) or the more recent EULAR/ACR 2019 criteria, which combine clinical features and immunologic findings to determine whether a patient meets the threshold for lupus diagnosis.

Recent advancements in deep learning have greatly improved medical imaging applications^24–29^. AI-based techniques have shown success across various healthcare tasks, including skin cancer detection, lung segmentation, brain disorder identification, pneumonia diagnosis, breast cancer detection, and fundus image analysis. Also, in other domains as dependencies for healthcare sector AI can be efficiently used, such drug discovery, misinformation about the diseases in social media and other vital tasks^7–15^.These AI-driven approaches help address challenges like RT-PCR test shortages and extended result processing times. The availability of publicly accessible medical image datasets enables researchers to apply AI techniques to identify patterns for automatic diagnosis^15–20^. Notably, individuals with both Lupus Erythematosus and pre-existing heart conditions face a greater risk of severe complications and mortality, suggesting a complex and not fully understood relationship between Lupus Erythematosus and cardiovascular health. Attempts to develop a universal diagnostic model for Lupus Erythematosus, irrespective of underlying health conditions, have been met with limited success, as shown in the practical analysis of this study^30^. Recognizing the importance of early diagnosis in cardiac care, this paper introduces a specialized model designed to enhance the accuracy of Lupus Erythematosus detection in patients with heart conditions. The objective is to improve diagnostic precision while minimizing delays, thereby aiding in the timely treatment of cardiac patients affected by Lupus Erythematosus. The heart’s electrical activity is captured through electrocardiogram (ECG) waveforms, offering essential insights into heart rhythm and electrical function. An ECG is made up of distinct elements, including P waves, QRS complexes, and T waves, each representing the depolarization and repolarization of the heart’s chambers. Conventional ECG monitoring involves placing electrodes on the skin using a 12-lead system. Important ECG parameters, such as the PR interval, QRS duration, QT interval, PR segment, and ST segment, are crucial for evaluating cardiovascular health. Computerized techniques are used to extract these temporal features from digitized ECG signals, facilitating faster and more accurate diagnoses. Identifying arrhythmias at an early stage is essential for managing cardiovascular conditions effectively.

This study proposes a specialized ensemble framework comprising two primary components. The initial module incorporates Residual Network (ResNet) blocks to derive critical features from ECG data, which are subsequently fed into a Long Short-Term Memory (LSTM) model to perform classification. To address class imbalance and enhance the quality of the training data, this stage integrates the Synthetic Minority Over-sampling Technique (SMOTE). The second subsystem presents an innovative pipeline that transforms ECG images into audio, facilitating the generation of Mel-spectrograms and advanced analysis using a fine-tuned Audio Spectrogram Transformer (AST). This audio-based approach uncovers more detailed temporal and spectral features, resulting in a more precise and interpretable classification compared to traditional techniques. Although deep learning holds significant potential for advancing medical diagnosis, several challenges hinder its widespread adoption. Key limitations include (1) the computational complexity and high energy demands of deep learning models, making deployment difficult in resource-constrained environments, (2) vulnerability to adversarial attacks, which raises security concerns in applications like autonomous systems, and (3) a lack of transparency, reducing trust in AI-driven decision-making. This study specifically addresses the issue of explainability by incorporating an explainable artificial intelligence (XAI) approach to enhance the interpretability of model predictions. The proposed method enables the investigation of how Lupus Erythematosus affects cardiac health, yielding meaningful observations regarding its influence on the cardiovascular system.

The structure of this paper is as follows: The Related Works section reviews existing literature on Lupus Erythematosus classification. The Proposed Model section outlines the classification approach introduced in this study. The Experimental Evaluation section presents the performance analysis of the proposed model. Finally, the Conclusion section summarizes the findings and implications of this research.

## Related works

We delve into a comprehensive examination and contrast of the most influential and widely cited studies that have developed deep learning models for Lupus Erythematosus detection, as presented in Table 1. The basis of our comparative study revolves around four key aspects:

(1) Preprocessing Techniques: This section explores the various data refinement strategies applied to manage the input datasets and boost model effectiveness. As Lupus Erythematosus are relatively sparse, these processes are essential for attaining accurate diagnostic outcomes.
(2) Key Contributions of the Studies: We extract and contrast the core ideas and significant outcomes of the examined research papers, highlighting the distinctive methodologies and perspectives each study contributes to the field.
(3) Reported Findings: A thorough evaluation of the performance metrics presented in each paper is performed, drawing attention to their respective results and what they signify for the ongoing development of deep learning applications in Lupus Erythematosus detection.

**Fig. 1:**
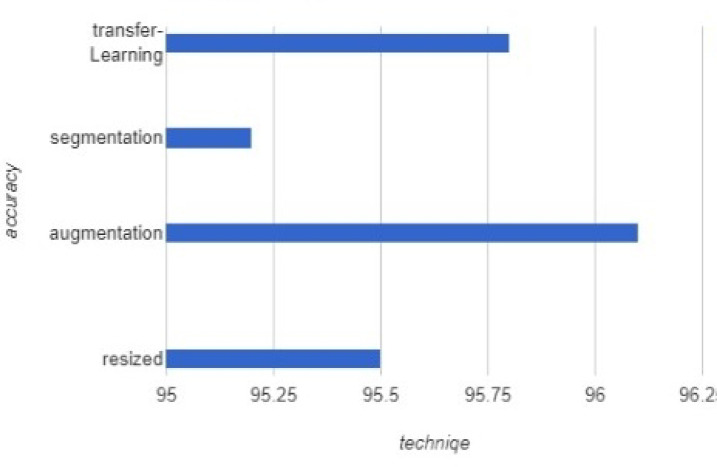
Comparative analysis of different pre-processing methods for datasets.

Given the novelty of the Lupus Erythematosus pandemic, the limited nature of available datasets necessitates thorough consideration of preprocessing techniques for optimal model performance. To better evaluate the impact of these preprocessing strategies, we offer a side-by-side comparison using the mean accuracy obtained across the ten most frequently cited studies utilizing each specific method. This analysis is represented in Fig. 1, providing a comprehensive overview of the relative performance of these techniques across the reviewed literature.

**Table 1:** Novel and comprehensive comparison among deep learning based diagnostic model.

Refs.	Pre-processing				Points	Accuracy
	Exclusions	Augmentation	Segmentation	Resize		
^1^	✓	–	–	–	A deep stacked architecture combining support vector machines (SVM-DSN) is developed to perform quantitative evaluation.	70%
^2^	–	–	–	✓	A dataset comprising 446 patients was assembled, along with corresponding clinical information. The models EfficientNet-B3 and ResNet-18 were applied for analysis in this work.	81.4%
^3^	✓	–	–	–	A recursive feature elimination method is applied to identify the key metabolites most relevant to distinguishing NPSLE. we construct a deep stacked network integrating support vector machines (SVM-DSN) for their quantitative assessment.	97.5%
^4^	✓		✓		a deep learning framework that combines deep neural networks with graph-based learning to detect genes associated with SLE. By extracting meaningful patterns from gene interaction graphs, the task of identifying SLE-linked genes is reformulated as a binary classification challenge, which is addressed using a fully connected neural layer	93.1%
^5^				✓	The performance of three convolutional neural network models—Dilated-CNN, Attention-Enhanced CNN (CNN-AM), and a conventional CNN—was assessed for their effectiveness in differentiating SLE-positive from SLE-negative instances using skin image data obtained from the DermNet collection.	96.7%
^6^			✓		A neural network model was developed using patient clinical information, and key features were assessed through an autoencoder framework.	89.5%
^34^			✓		The network leverages Bi-LSTM gating to capture long-term contextual dependencies in ECG signals. A residual deformable convolution module is integrated, enhancing local feature extraction through adaptive receptive fields.	86.3%
^35^	✓				This study introduces a novel approach by tailoring CNN architectures and strategically integrating GRU capabilities to effectively compress and denoise ECG signals.	90.1%
^36^	✓				Key features are selected through recursive feature elimination (RFE), after which disease prediction is carried out using a hybrid mutation-driven swarm intelligence model integrated with an attention-enhanced GRU network.	95.42%
^37^	✓				A convolutional neural network was developed using ECG data from the cardiology clinic at Boston Children’s Hospital, with an even 50/50 split for training and testing to predict 5-year mortality.	95%

### Discussion

Deep learning models for Lupus Erythematosus diagnosis face a major challenge due to the limited availability of Lupus Erythematosus samples in public datasets, primarily because of the pandemic’s recent emergence. This scarcity can negatively impact model performance^21,23^. However, Model 9 demonstrates exceptional sensitivity and specificity compared to other models, despite being trained on a dataset with very few Lupus Erythematosus samples. This success is largely due to the implementation of data augmentation, an effective technique that expands the dataset size by generating additional variations of existing samples. By addressing class imbalance, data augmentation significantly enhances model performance on limited datasets. Furthermore, previous studies^1,2^ have shown that transfer learning can also produce impressive results. Transfer learning is particularly well-suited for training on small datasets, such as those used for Lupus Erythematosus detection. Given these findings, it is essential for researchers to further investigate specialized techniques that mitigate the challenges associated with limited Lupus Erythematosus datasets. A strong focus on transfer learning and data augmentation strategies can enhance the robustness and generalization capabilities of deep learning models for Lupus Erythematosus diagnostics. The whole approach is presented in.

## Methodology

### Dataset generation

This research concentrates on developing a unique deep learning-based model for diagnosing Lupus Erythematosus in heart patients. Unlike previous methods, which sought to create broad models for the general population, our approach is specifically tailored to address the unique challenges faced by individuals with pre-existing heart conditions, who are at a significantly higher risk of severe complications from Lupus Erythematosus. This focused strategy aims to improve diagnostic precision and enhance care for this vulnerable group.

To construct our dataset, we use electrocardiogram (ECG) images, which provide an accurate reflection of the heart’s electrical activity. ECG is commonly employed to identify heart abnormalities and assess cardiac health. Researchers in China carried out a cross-sectional study to assess how frequently ECG irregularities occur among individuals with SLE, and to identify contributing factors through the application of machine learning techniques^31^, which demonstrates that clinical severity in Lupus Erythematosus patients can be evaluated through the analysis of troponin levels and electrocardiographic irregularities. The proposed model aims to determine the Lupus Erythematosus status—positive or negative—of individuals with pre-existing heart conditions by analyzing their ECG data. Where available, we have documented the demographic characteristics of the patients in our dataset. The age range of the patients spans from 25 to 75 years, with a median age of 43. Approximately 58% of the subjects are female and 42% are male. The majority of the patients originate from regions in East Asia and the Middle East, as reflected in the source databases. However, specific ethnicity data was not included in the original dataset.

**Fig. 2:**
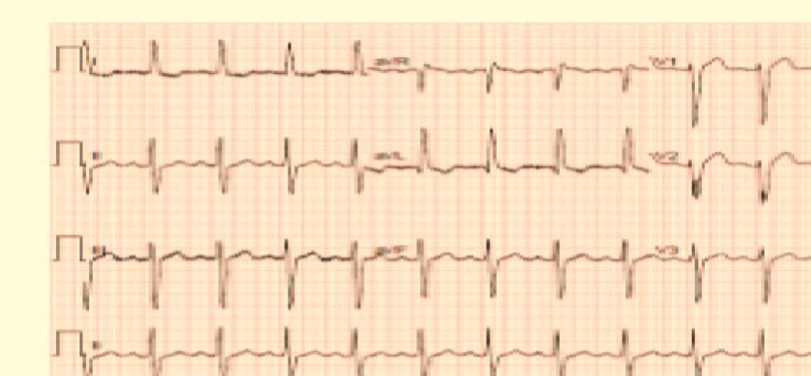
ECG for cardio patient and Lupus Erythematosus.

**Fig. 3:**
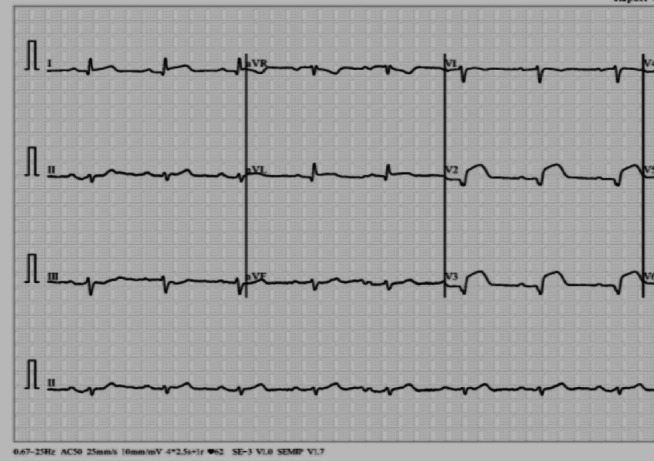
ECG for cardio patient with non-Lupus Erythematosus.

The dataset utilized comprises approximately 250 ECG images from confirmed Lupus Erythematosus cases, 77 images from patients diagnosed with Myocardial Infarction, 203 samples from individuals with a prior history of Myocardial Infarction, and 504 images from subjects exhibiting abnormal heart rhythms. Additionally, the dataset consists of 859 ECG images from individuals with normal heart function. For the purposes of this study, we organize the dataset into two primary categories: ECG images from heart patients with a confirmed Lupus Erythematosus diagnosis and heart patients that uninfected with Lupus Erythematosus, samples for the two classes are shown in Figs. 2 and 3, respectively. To ensure the accuracy and relevance of our model’s predictions, we collaborated closely with a cardiac diseases expert during the dataset updating process. This collaboration resulted in a meticulous review and refined categorization of the ECGs of Lupus Erythematosus cases into two distinct groups: heart patients with a positive Lupus Erythematosus case and heart patients with a non-Lupus Erythematosus case, as shown in Figures^2,3^.

The complete dataset of ECG samples was randomly divided into 80% for training and 20% for testing. The split was stratified to maintain class balance between LE-positive and LE-negative cases.

### The proposed model

**Fig. 4:**
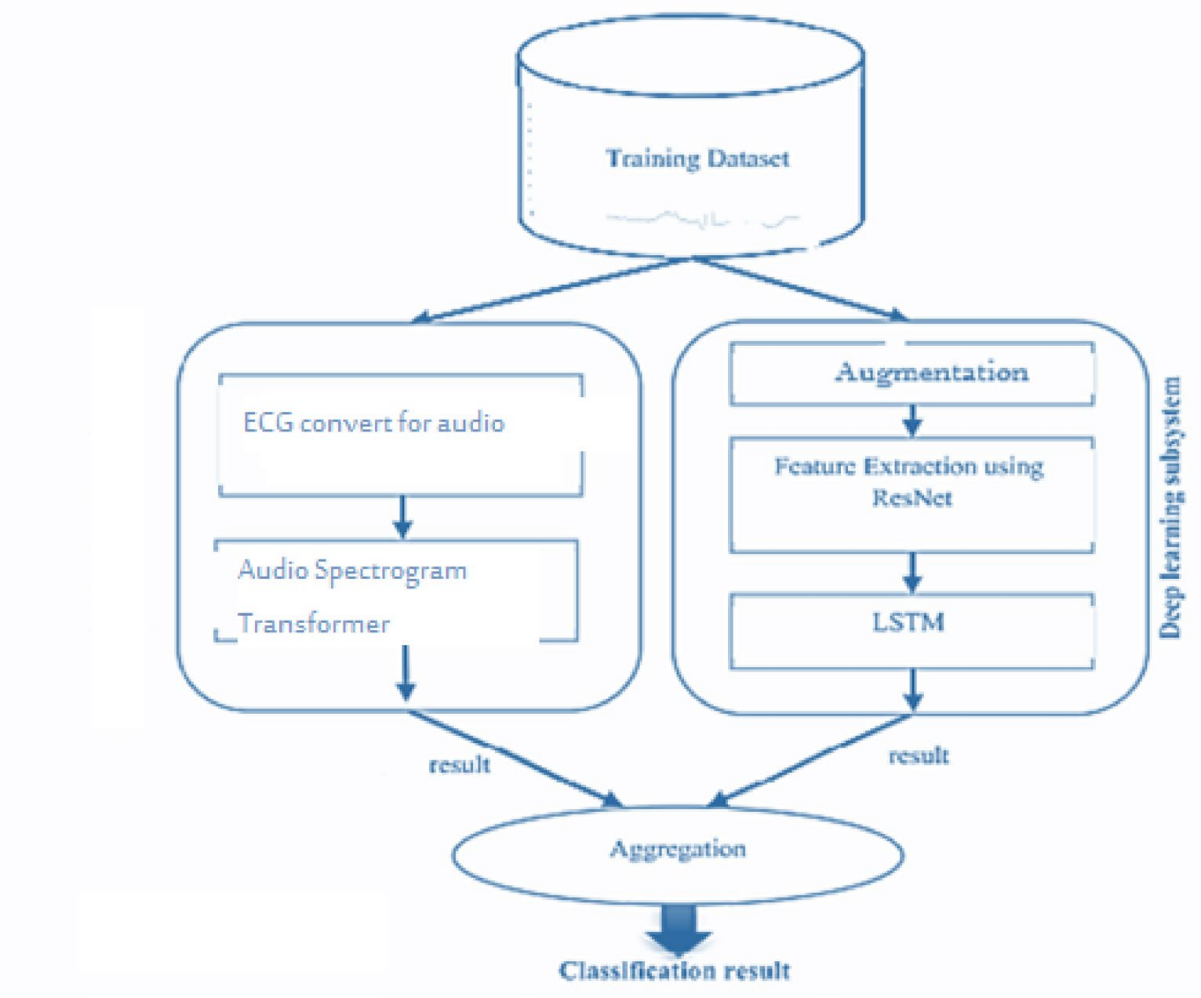
Architecture of our proposed models.

Our proposed model introduces an efficient Lupus Erythematosus diagnostic system tailored for heart patients, incorporating two autonomous subsystems that operate simultaneously. These subsystems analyze the patient’s condition and generate a final diagnosis through an aggregation method. To maintain a balanced training dataset, the SMOTE data augmentation technique is applied. The first subsystem utilizes two deep learning architectures—ResNet and LSTM—for feature extraction and classification. Meanwhile, the second subsystem extracts key ECG signal features, including peak, temporal, and morphological characteristics, which are then classified using a Random Forest classifier. By integrating deep learning and traditional machine learning techniques, our approach leverages the strengths of both methodologies. To produce the final diagnosis, outputs from both subsystems undergo weighted aggregation. Figure 4 provides an overview of the classification process. During training, the dataset is processed by both subsystems, and in the testing phase, classification occurs in parallel, leading to the final result. Figure 9 outlines the step-by-step implementation of our whole approach. In this approach, the inputs comprise ECG signals and their associated labels, while the outputs contain essential evaluation metrics. The methodology in both subsystems is structured into two main stages: feature extraction and data classification.

#### The first subsystem: lupus erythematosus detection for heart patient

**Fig. 5:**
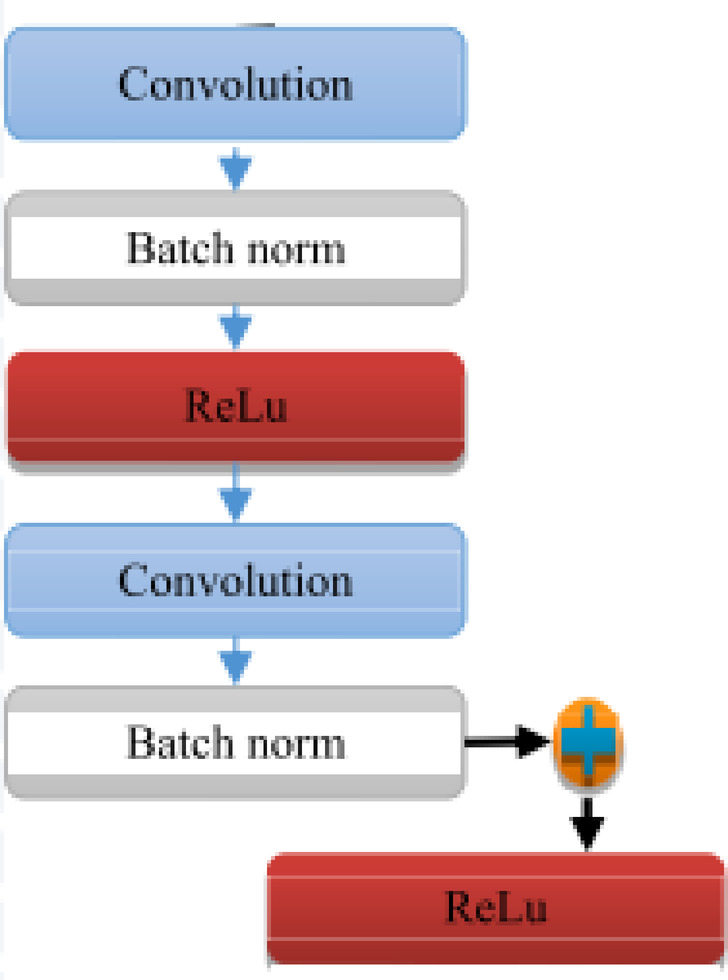
The architecture of Resnet.

The first subsystem, designed for cardiac arrhythmia detection, integrates a ResNet architecture with two LSTM networks. The process begins by converting the input ECGs into a two-dimensional matrix, which is subsequently processed through three consecutive ResNet blocks to extract key features. These extracted features are then passed to two LSTM networks, where the first acts as an encoder and the second as a decoder. The output from the second LSTM network is passed through a Softmax layer for classification of the patient’s condition, as defined in Eq. 1. Figure 5 demonstrates the ResNet block used for feature extraction within this subsystem. This block comprises three layers, incorporates a skip connection, and utilizes ReLU activation functions to enhance learning efficiency.

1 $$\begin{gathered} Ou{t_{(xl+1)}}\,=\,f{\text{ }}\left( {{\text{ }}{x_{l+1}},{\text{ }}\{ {W_l}^{{(i)}}\} } \right)\,+\,{x_l}\,Where{\text{ }}f{\text{ }}\left( {{\text{ }}{x_{l+1}},{\text{ }}\{ {\text{ }}{W_l}^{{(i)}}\} } \right) \hfill \\ \quad \quad \quad \,={\text{ }}{\left( {{\text{ }}{W_l}^{{(2)}}} \right)^T}\sigma (BN({\left( {{\text{ }}{W_l}^{{(1)}}} \right)^T}\sigma \left( {BN\left( {{x_l}} \right)} \right))) \hfill \\ \end{gathered}$$

In this context, W(i) refers to the i-th weight within the l-th module, where i takes values of 1 or 2. The activation function used is denoted as σ (specifically ReLU), and BN represents the operation for batch normalization. The variable x_l_ denotes the input features for the l-th module. The function f: x_l_ → x_l+1_ describes the transformation from the input vector x_l_ to the output vector x_l+1_ in the fundamental vect.

**Fig. 6:**
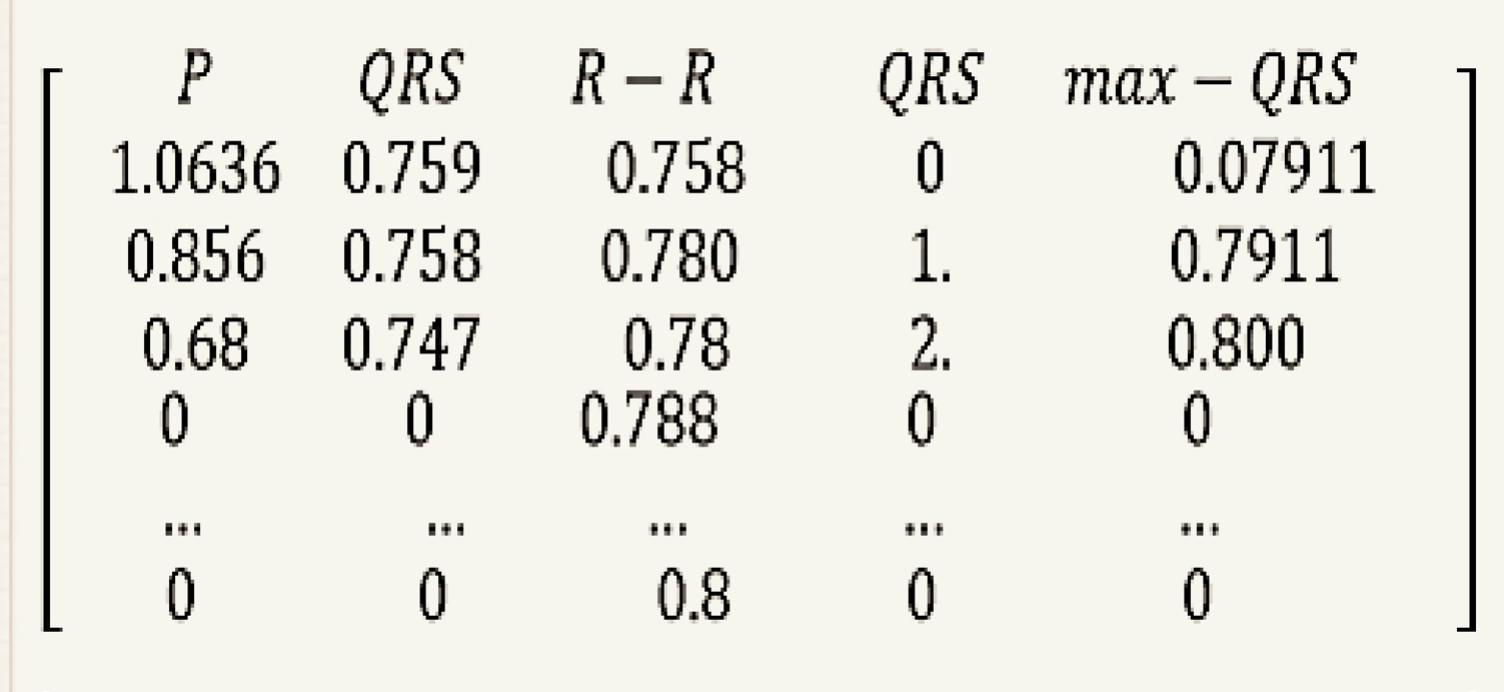
One of the matrices that we transformed from its ECG sample.

Figure 4 demonstrates the procedure for extracting features from each ECG signal. First, the ECG data is converted into a matrix initialized with zeros, based on the maximum length of the extracted feature sets. The matrix dimensions are set to N * 5, where N represents the length of the largest feature set. The features within the matrix are arranged as follows: Column 0 includes all R-R intervals; Column 1.

includes all P wave values; Column 2 contains all QRS values; Column 3 holds all R-R intervals; Column 4 records all indices of QRS peaks; and Column 5 represents the highest amplitude for each QRS complex, as illustrated in Fig. 6.

**Table 2:** Layers Pf the ResNet model.

Layer Name	Kernal Size	Input shape	Output Shape
Convolutional	2 * 1	10 * 28	10 * 1 * 32
Convolutional	2 * 1	10 * 1 * 32	10 * 1 * 32
Max Pooling	2 * 1	10 * 1 * 32	8 * 1 * 32
Convolutional	2 * 1	5 * 1 * 64	5 * 1 * 64
Convolutional	2 * 1	5 * 1 * 64	5 * 1 * 64
Max Pooling	2 * 1	5 * 1 * 64	3* 1 * 64
Convolutional	2 * 1	3 * 1 * 64	3 * 1 * 128

The extracted matrix is then passed through three ResNet blocks, each consisting of 18 layers, to further enhance feature extraction from the input data. Max pooling is applied after the first two ResNet blocks to simplify the model, highlight essential features, and increase network efficiency, as shown in Table 2. However, due to the reduced dimensionality, pooling is omitted following the third block. The extracted features are then used for the final classification process.

The network architecture consists of three ResNet blocks, each containing two convolutional layers, resulting in a total of six convolutional layers. Two Max Pooling layers are included, though pooling is omitted in the final block. The kernel sizes, pooling methods, and stride values are optimized for the two-dimensional input data, as outlined in Table 2. Experimental tests were conducted to determine the optimal learning rate and number of training epochs. In this subsystem, traditional LSTM units (RNNs) are substituted with bi-directional RNN (BiRNN) units. Unlike standard RNNs, which process data in one direction, BiRNNs analyze the input in both forward and reverse directions, providing access to both past and future information simultaneously. The BiRNN is composed of two distinct networks: one processes the input sequence in its natural order (t = 1, 2, …, T), and the other processes it in reverse order (t = T, T-1, …, 1). The final output of the BiRNN is obtained by combining the weighted results from both networks, as outlined in Eqs. 2–4.

2 $$\:\overrightarrow{{h}_{t}}=\:tanh\:(\overrightarrow{Wx}t\:+\:\overrightarrow{V\:}\overrightarrow{\:h\:{\:}_{t-\:1}\:}+\overrightarrow{b})$$

3 $$\:\overleftarrow{{h}_{t}}=\:tanh(\overleftarrow{Wx}+\:\overleftarrow{V}\:\overleftarrow{h\:{\:}_{t-\:1}\:}+1\:+\overleftarrow{b})$$

4 $$\:yt\:=\:\left(U\right[\overrightarrow{{h}_{t}}\:,\:\overleftarrow{{h}_{t}}\:]\hspace{0.17em}+\hspace{0.17em}{b}_{y})$$

The equation presented incorporates the hidden state alongside the bias from the forward network, with the hidden state and the backward network bias corresponding to each other. In the BiRNN, the inputs and outputs are denoted as ‘a’ and ‘b’, respectively. The decoder network generates an output sequence for each time step, functioning similarly to the encoder network. There are multiple architectural designs for ResNet blocks, and the specific ResNet block shown in Fig. 5 consists of six layers. Research has shown that the ResNet architecture effectively mitigates the issue of gradient vanishing, which is why it was selected for feature extraction.

**Fig. 7:**
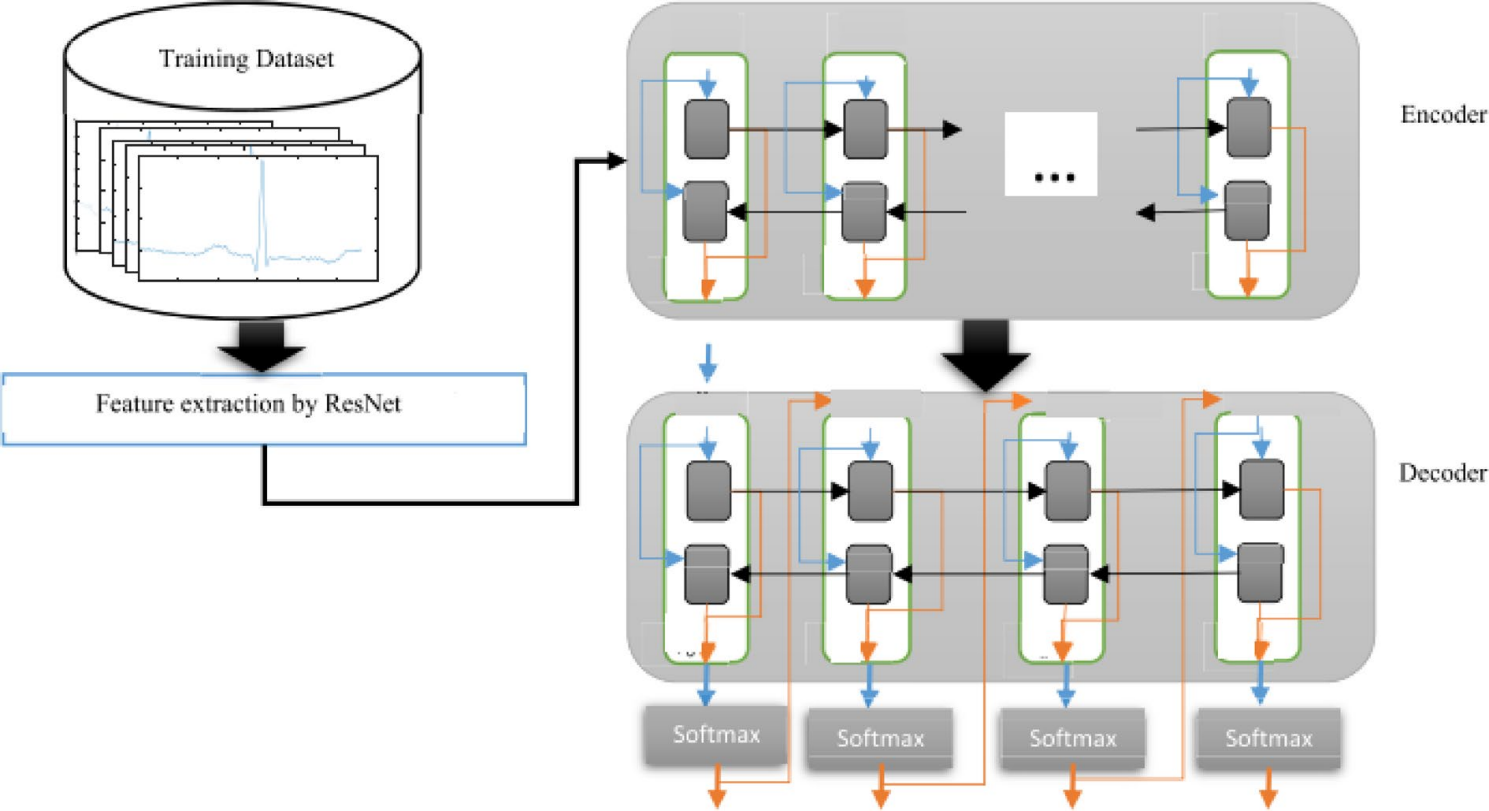
LSTM components for features classification.

The features extracted from the ResNet blocks are then passed into encoder-decoder LSTM networks, which process the data and perform classification tasks, as depicted in Fig. 7. At the end of each LSTM, a Softmax layer is included to calculate the probability of each signal being assigned to its corresponding class.

The ResNet and LSTM components used in this subsystem were trained from scratch. We initialized the weights using the He Normal (Kaiming) initialization method to ensure stable gradient propagation in deep networks. The models were optimized using the AdamW optimizer with a learning rate of 1 × 10⁻⁴ and weight decay of 0.01. These hyperparameters were selected through grid search based on validation loss performance, aiming to minimize overfitting while ensuring fast convergence.

#### The second subsystem: ECG signal conversion to audio

The analysis of Electrocardiogram (ECG) signals is crucial for diagnosing various cardiac conditions. Traditional methods of analyzing ECG data directly in the time domain may not capture subtle variations, especially those induced by Lupus Erythematosus infection. The transformation of ECG signals into audio representations is driven by the rhythmic and quasi-periodic nature of ECG waveforms, which are structurally analogous to acoustic signals. By converting the signal to audio and then into a Mel-spectrogram, we enable rich time–frequency analysis that captures both morphological and spectral characteristics of cardiac activity. This representation is especially valuable for identifying subtle abnormalities induced by Lupus Erythematosus. Furthermore, it allows us to leverage pretrained models like AST, originally designed for audio classification, through transfer learning—yielding improved feature extraction and classification even on limited medical datasets. This fusion of biomedical signal processing with modern audio-based deep learning opens new avenues for enhanced ECG analysis.

In this study, we propose a novel approach where ECG signals are first converted from images into audio representations, then transformed into Mel-spectrograms. These audio representations are used to train deep learning models capable of identifying abnormalities, leveraging advanced models commonly used for speech and audio classification.

**I. ECG Signal Extraction from Image**.

ECG signals are often available in the form of images, such as scanned or photographed ECG plots. To convert these images into time-series ECG waveforms, the first step involves preprocessing the image. Initially, the raw ECG image is converted into grayscale to reduce complexity, followed by applying Gaussian blur and adaptive thresholding to remove background noise and emphasize the ECG signal. This process highlights the ECG waveform, which is then extracted by detecting the largest contour in the image. The position of the waveform along the x-axis (time) is determined by tracking the contour across the image, and for each column in the image, the position of the contour is used to extract the corresponding ECG amplitude, thus forming a 1D time-series signal. Mathematically, the extracted ECG waveform can be represented as x(t), where t denotes discrete time f in the ECG signal. While the image-to-audio pipeline introduces a novel diagnostic perspective, we recognize that it may also introduce artifacts during signal extraction. These can arise from background noise, grid lines, or imperfect contour tracking. To reduce such issues, we applied preprocessing steps including grayscale conversion, Gaussian blur, and adaptive thresholding. The largest contour, assumed to represent the primary ECG trace, was selected to minimize interference. We also manually inspected samples during development to confirm waveform fidelity. Furthermore, the AST model’s robustness—enhanced through Spec Augment and fine-tuning—helps accommodate small inconsistencies in the audio input. These combined strategies reduce the risk of error propagation due to preprocessing artifacts.

**II. Audio Conversion of ECG Signal**.

Once the raw ECG waveform is extracted, it is normalized and converted into a time-domain audio waveform. This normalization step ensures that the audio signal adheres to standard formats used in audio processing. Normalization is applied to scale the ECG signal amplitude to the range [–1, 1], which is the standard for audio waveform processing. This ensures consistent loudness levels and prevents clipping during audio conversion. The choice of an 8000 Hz sampling rate is based on established practices in biomedical audio signal processing, particularly in heart sound and phonocardiogram analysis, where the frequency content of interest typically lies below 4 kHz. Sampling at 8000 Hz satisfies the Nyquist criterion while minimizing unnecessary computational overhead compared to higher rates such as 16–22 kHz, which are better suited for speech signals. This rate provides a good trade-off between fidelity and efficiency for ECG-derived audio. The normalization is performed using the following formula:

$$\:{x}_{norm}\left(t\right)=\frac{x\left(t\right)-\mu\:\left(x\right)}{max(\mid\:x(t)\mid\:)}$$

where µ(x) is the mean of the ECG signal and max(∣x(t)∣) is the maximum absolute value of the signal. After normalization, the ECG waveform is resampled at a standard audio sampling rate (e.g., 8000 Hz), converting the ECG signal into a continuous audio signal that can be saved as a .wav file. This audio format enables the use of sound-based deep learning models for further analysis.

**III. Final Generation for Audio representation**.

The next step involves converting the audio waveform into a Mel-spectrogram, a widely-used representation in audio processing that provides a time-frequency analysis of the signal. The process begins with applying the Short-Time Fourier Transform (STFT) to the audio waveform to extract its frequency content over time. The STFT is calculated as follows:

$$\:\text{S}(\text{f},\:\text{t})=\sum\:_{n=-\infty\:}^{\infty\:}\text{x}\left(\text{n}\right)\text{w}\left(\text{n}-\text{t}\right){\text{e}}^{-\text{j}2{\uppi\:}\text{f}\text{n}}\:$$

where x(n) represents the audio signal at time n, and w(n − t) is the window function applied to the signal. f denotes the frequency, and t represents time. Following this, the frequency content is mapped to the Mel scale, which is a logarithmic scale designed to approximate the frequency perception of the human ear. The Mel scale is given by:

$$\:M\left(f\right)\hspace{0.17em}=\hspace{0.17em}{2595\:\text{l}\text{o}\text{g}}_{10}\:(1+\frac{f}{700})$$

**Fig. 8:**
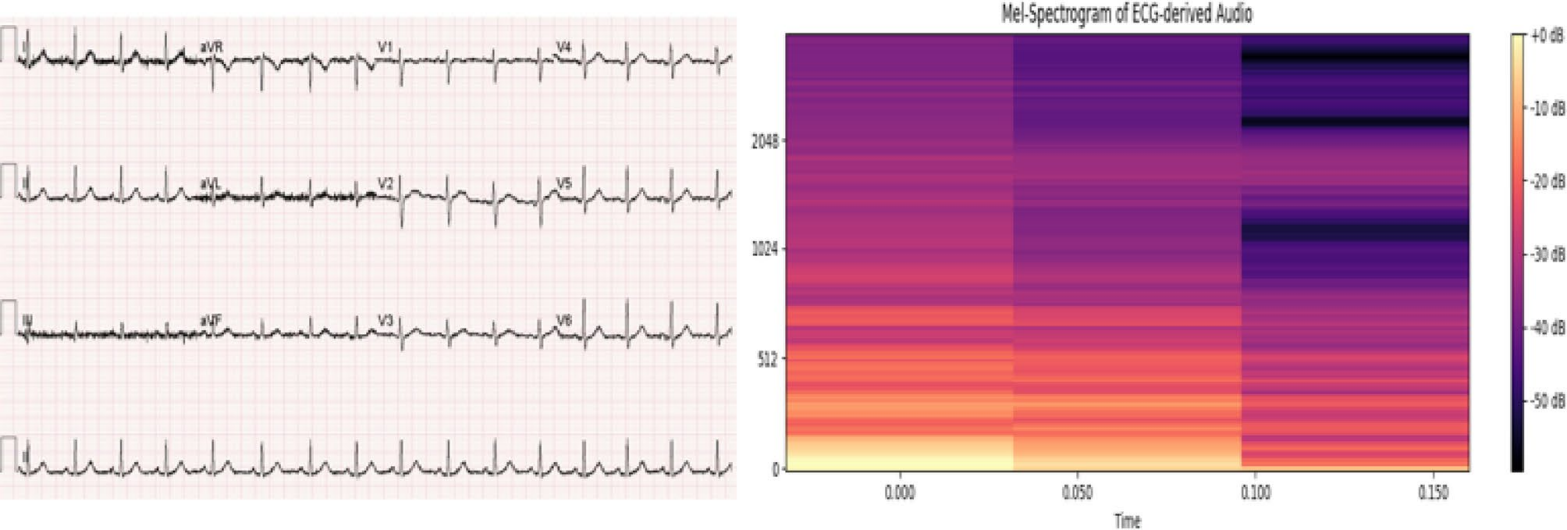
A sample for an ECG in the dataset and generated corresponding spectrogram.

Subsequently, the Mel-spectrogram is converted to decibels (dB) to make the magnitude of frequencies more interpretable. The resulting Mel-spectrogram captures both the temporal and frequency components of the ECG signal, which are essential for identifying subtle cardiac abnormalities, as shown in Fig. 8.

**IV. Classification Using Audio Spectrogram Transformer (AST)**.

To enhance the classification of ECG signals, particularly for detecting subtle cardiac abnormalities associated with Lupus Erythematosus, we employed the Audio Spectrogram Transformer (AST), a transformer-based architecture initially developed for audio classification tasks. AST has demonstrated state-of-the-art performance on large-scale audio datasets such as AudioSet, making it a compelling choice for time-frequency analysis of biomedical signals. The model’s ability to extract both local features and long-range temporal dependencies allows it to effectively analyze the complex variations present in ECG signals when represented as Mel-spectrograms.

Prior to model training, ECG signals were transformed from their raw image form into one-dimensional time-series waveforms using a pipeline involving grayscale conversion, Gaussian blurring, adaptive thresholding, and contour detection. These time-series signals were subsequently converted into audio waveforms, which were then transformed into log-Mel spectrograms using a Short-Time Fourier Transform (STFT). The STFT was configured with a sampling rate of 22,050 Hz, a window size of 1,024, a hop length of 512, and 128 Mel frequency bands. The resulting spectrograms were resized to 224 × 224 pixels and converted into three-channel images to match the input requirements of the AST model. These spectrograms served as input to the AST model, which was fine-tuned for our specific classification task. We utilized the pretrained AST model released by the Massachusetts Institute of Technology (MIT), originally trained on the AudioSet corpus. The model’s classification head was replaced with a custom fully connected neural network designed to accommodate our three output classes: normal, abnormal, and Lupus Erythematosus affected ECG signals. During training, we initially froze the transformer encoder layers and trained only the classifier to stabilize convergence. In later stages, the encoder layers were unfrozen for full fine-tuning, allowing the model to adapt to the domain-specific features of ECG-derived spectrograms.

The model was trained using the AdamW optimizer with a learning rate of 2 × 10⁻⁵ and a weight decay of 0.01. A batch size of 16 was used, and training proceeded for 50 epochs. We applied categorical cross-entropy loss and employed early stopping and model checkpointing based on validation loss. To improve generalization, data augmentation was implemented through SpecAugment, which involved applying random masking on time and frequency dimensions of the spectrograms. In addition to classification performance, we emphasized model interpretability. Attention maps were extracted from the AST architecture to visualize the spectro-temporal regions that contributed most significantly to each classification decision. These maps revealed that the model consistently attended to regions corresponding to waveform irregularities or frequency distortions commonly associated with LUPUS ERYTHEMATOSUS 19-induced abnormalities. Figure 14 presents an attention heatmap overlaid on a Mel-spectrogram from a LUPUS ERYTHEMATOSUS affected ECG sample, highlighting the activated areas influencing the prediction. Despite AST’s relatively large architecture, the model maintained efficient inference times when run on GPU hardware. For future deployment in resource-constrained environments such as mobile health monitoring devices, model optimization techniques including pruning, quantization, and knowledge distillation will be explored. Overall, the AST model significantly outperformed conventional CNN and CRNN architectures in terms of classification accuracy, precision, recall, and F1-score, demonstrating its suitability for audio-based ECG analysis.

**Fig. 9:**
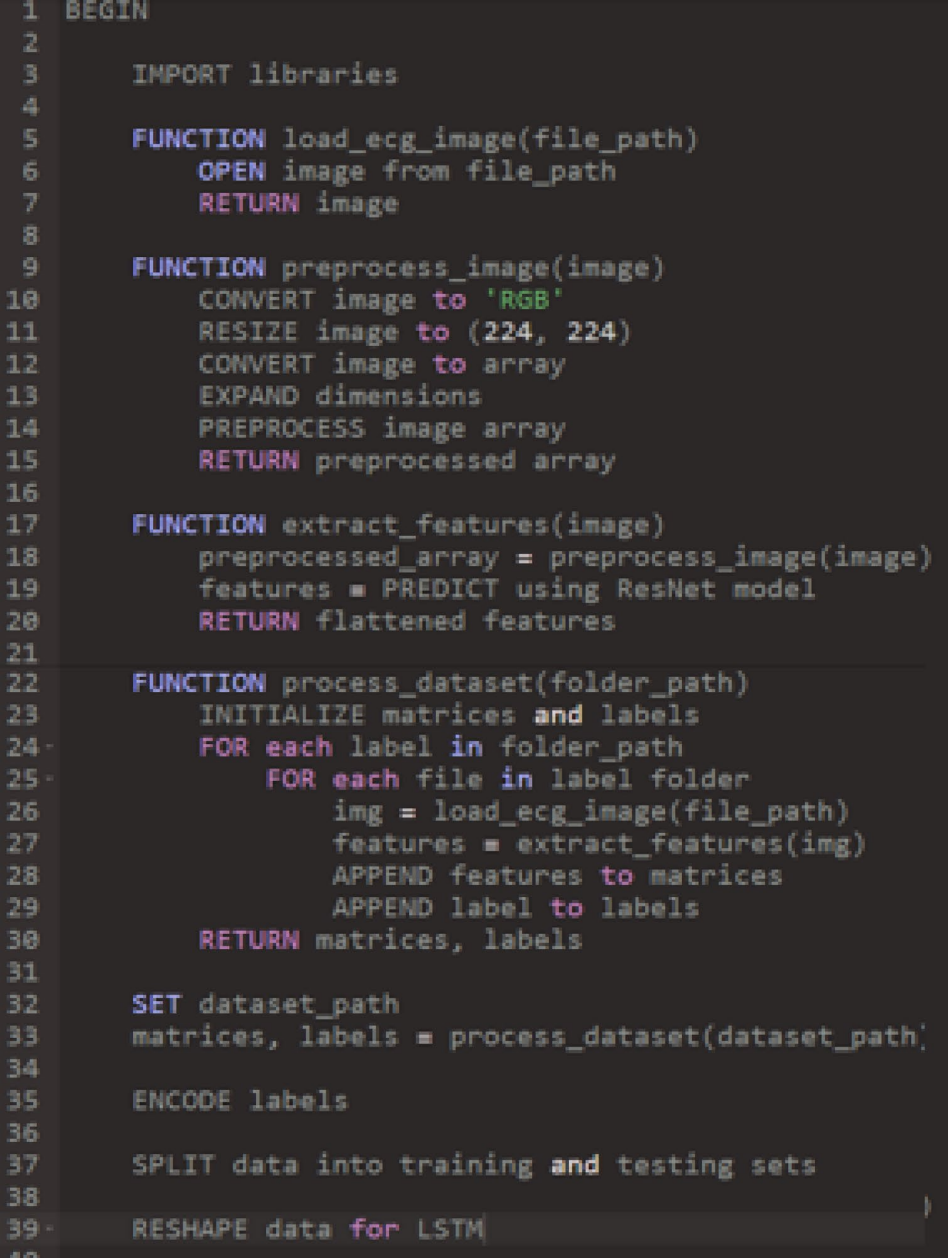
The pseudocode of our algorithm.

The pretrained AST model’s encoder weights were initially frozen and later unfrozen for full fine-tuning. Weight optimization was performed using the Adam optimizer with a learning rate of 2 × 10⁻⁵ and a weight decay of 0.01. These values were chosen empirically via cross-validation to balance training stability and generalization (Fig. 9).

### The explainable recent approach

The difficulty in comprehending the operations of black-box models poses an obstacle to the smooth integration of deep learning in medical imaging. In healthcare, where trust between clinicians and patients is essential, transparency and dependability are vital. Moreover, deep learning models hold the potential to reveal biologically significant patterns by identifying features that traditional diagnostic methods might miss.

The ability to derive meaningful insights from deep learning models relies on the interpretability and transparency of their decision-making processes. When the reasoning behind a model’s predictions is unclear, it raises concerns about the level of trust in its outcomes. To enhance explainability, two widely used techniques are SHapley Additive exPlanations (SHAP) and heatmaps. SHAP values help interpret a model’s decisions by quantifying the contribution of each feature to its predictions. These values indicate how much a specific feature influences the outcome, both at the individual prediction level and across the entire model. SHAP summary plots visualize the distribution of feature impacts, offering deeper insights into model behavior. On the other hand, heatmaps use color-coded representations to display patterns, correlations, and value distributions within a dataset. They provide an intuitive way to assess relationships between features, where darker shades typically indicate stronger correlations and lighter shades represent weaker associations. A comparison of these two approaches is presented in Table 3.

**Table 3:** Comparison between heatmap and SHAP values methodologies.

Feature	SHAP Values	Heatmaps
Purpose	Explain feature contributions to predictions	Visualize data patterns or relationships
Output	Numeric values showing feature impact	Color-coded grid representing values
Interpretation	Detailed understanding of model behavior	Quick visual assessment of data trends
Common Use	Model interpretability	Correlation analysis, data patterns

In our study, we tackle this issue by using a heatmap technique, specifically the gradient-weighted class activation mapping method, known as Grad-CAM. This approach is both class-specific and effective at identifying relevant regions within an image. Grad-CAM works by utilizing gradients to assign scores to different parts of an image based on a model’s evaluation of a given category. As shown in previous research^32^, Grad-CAM has outperformed other explainable methods, such as CAM and Grad-CAM++. As a result, we have opted to use Grad-CAM to interpret our model’s decisions and emphasize the key features in medical images, particularly bite marks in our case. This decision reflects our dedication to ensuring the transparency and interpretability of our deep learning-based diagnostic model within the medical imaging field.

#### Experimental evaluation

All experiments were conducted on a system equipped with an NVIDIA RTX 3090 GPU (24GB), Intel Core i9-12900 K CPU, and 64GB RAM. Model training was implemented in Python using PyTorch 2.0 and Librosa for signal processing. Training time per fold ranged from 15 to 30 min per subsystem.

Hyperparameters such as learning rate, batch size, and number of epochs were optimized through grid search using the validation set. For the ResNet-LSTM subsystem, the optimal configuration included a learning rate of 1e − 4, batch size of 32, and 25 epochs. For the AST-based audio subsystem, a learning rate of 2e − 5, batch size of 16, and 50 epochs produced the best results. Regularization was applied using weight decay (0.01) and dropout (0.3) where applicable.

#### Confusion matrix

**Fig. 10:**
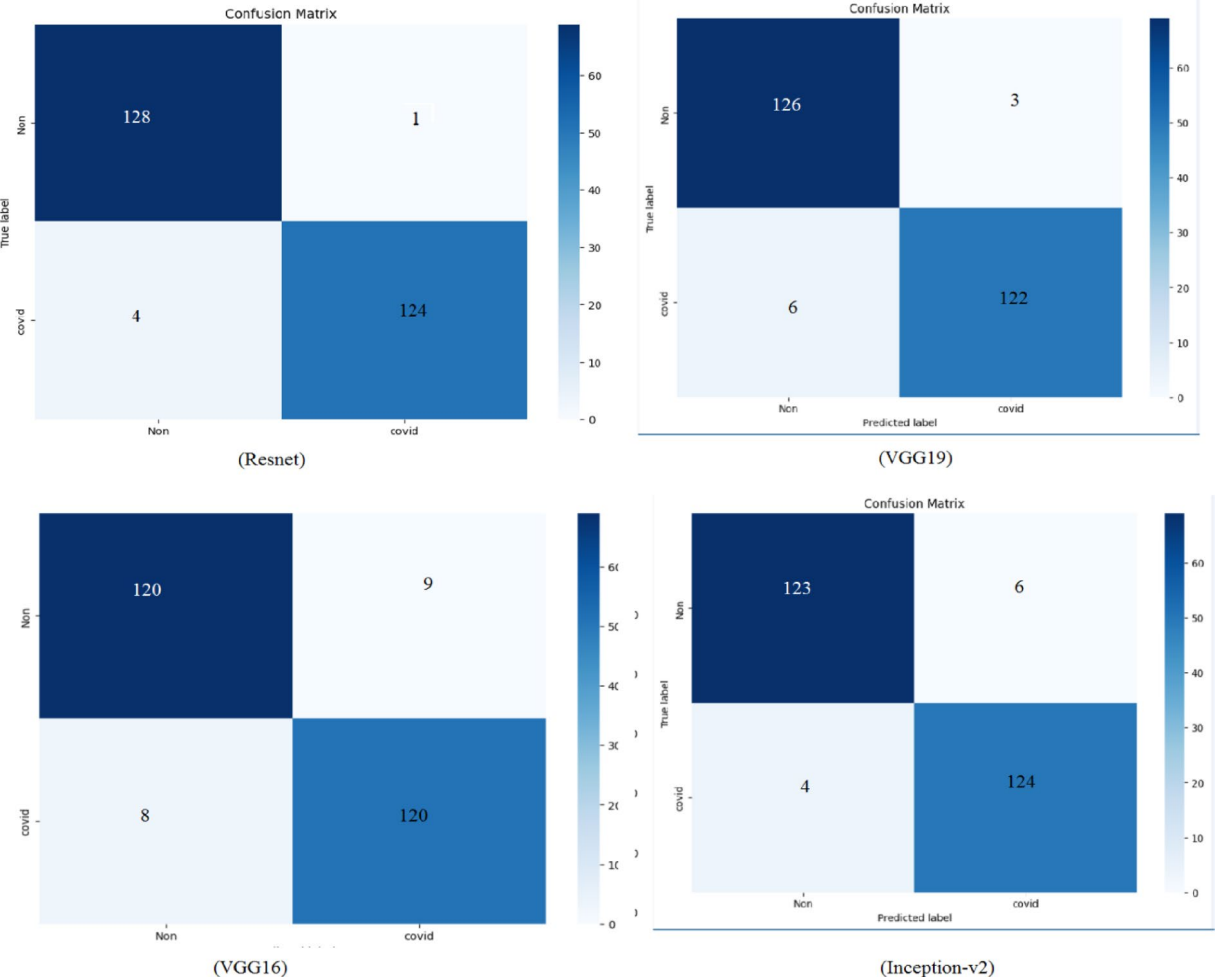
Confusion matrices of our algorithm with different models for feature extraction.

The confusion matrix comprises four factors: True Positive (TP), False Positive (FP), True Negative (TN), and False Negative (FN). In this matrix, the rows represent the ‘Real class values,’ while the columns denote the ‘Predicted class values,’ serving as an evaluative tool for the model’s efficiency. We have trained different models for the feature extraction task. We found that Resnet model achieved the best results as shown in Fig. 10. According to the confusion matrix generated for the validation dataset, our proposed model achieved a sensitivity of 99.5% and specificity of 97%. Sensitivity measures the proportion of True Positives (TP), or the number of instances in which the model correctly identifies Lupus Erythematosus patients among the actual Lupus Erythematosus patients. Specifically, from the 129 Lupus Erythematosus patients in the test dataset, our model accurately identified 128, resulting in a sensitivity of 99.2%. This demonstrates that our model can diagnose Lupus Erythematosus in patients with only a 0.08% error rate. On the other hand, specificity reflects the proportion of True Negatives (TN), or the number of instances where the model accurately predicts non- Lupus Erythematosus patients out of the actual non- Lupus Erythematosus patients. In the validation dataset, from the 128 non- Lupus Erythematosus patients, the model correctly predicted 124, yielding a specificity of 97%.

By calculating the overall accuracy based on the confusion matrix, we achieved an accuracy of 98.05%. This thorough evaluation, accounting for both sensitivity and specificity, highlights the strong performance and dependability of our diagnostic model in accurately distinguishing between Lupus Erythematosus and non- Lupus Erythematosus cases.

#### Training and validation accuracy

**Fig. 11:**
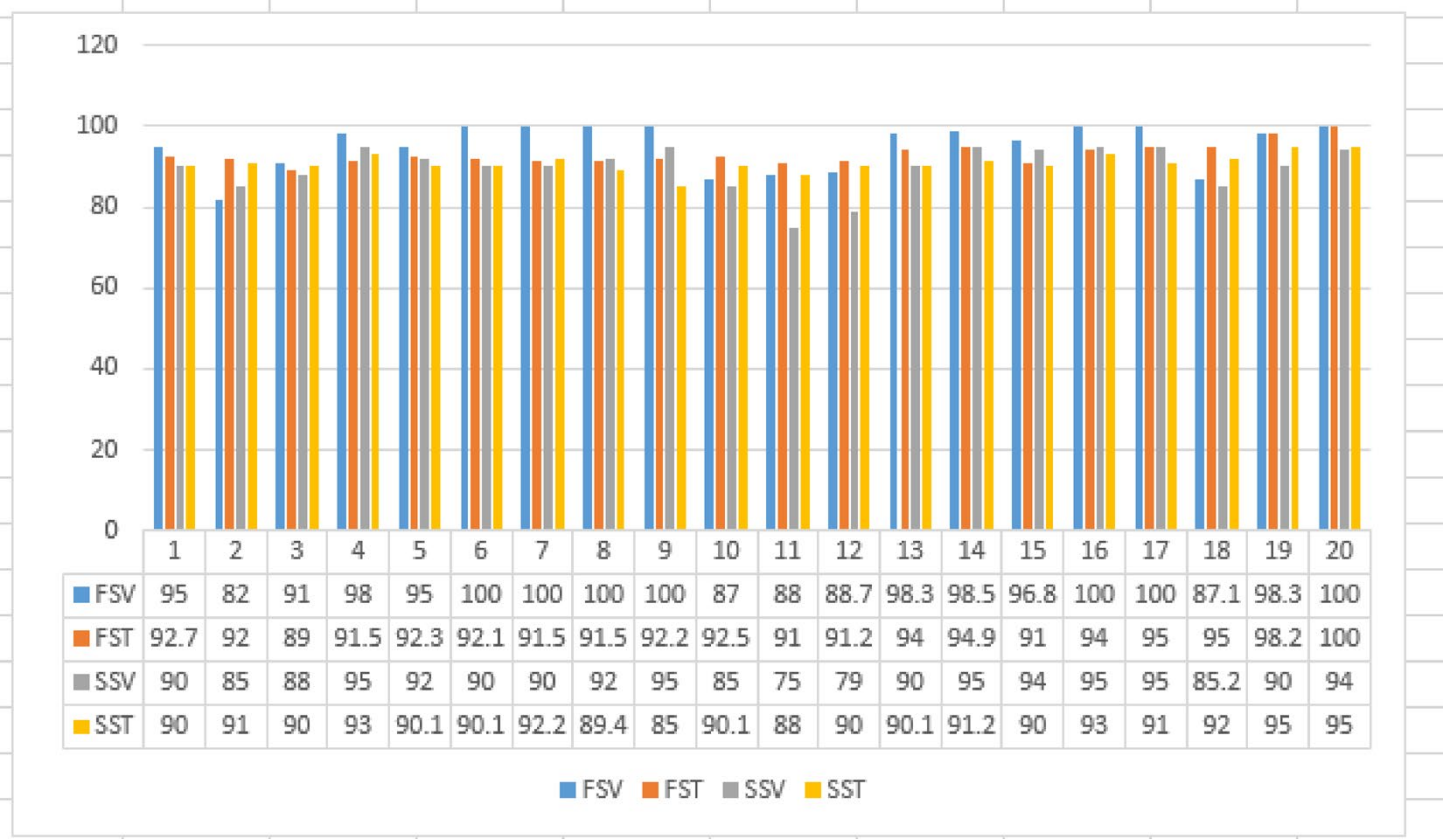
Training and validation accuracy for the two sub-systems.

**Fig. 12:**
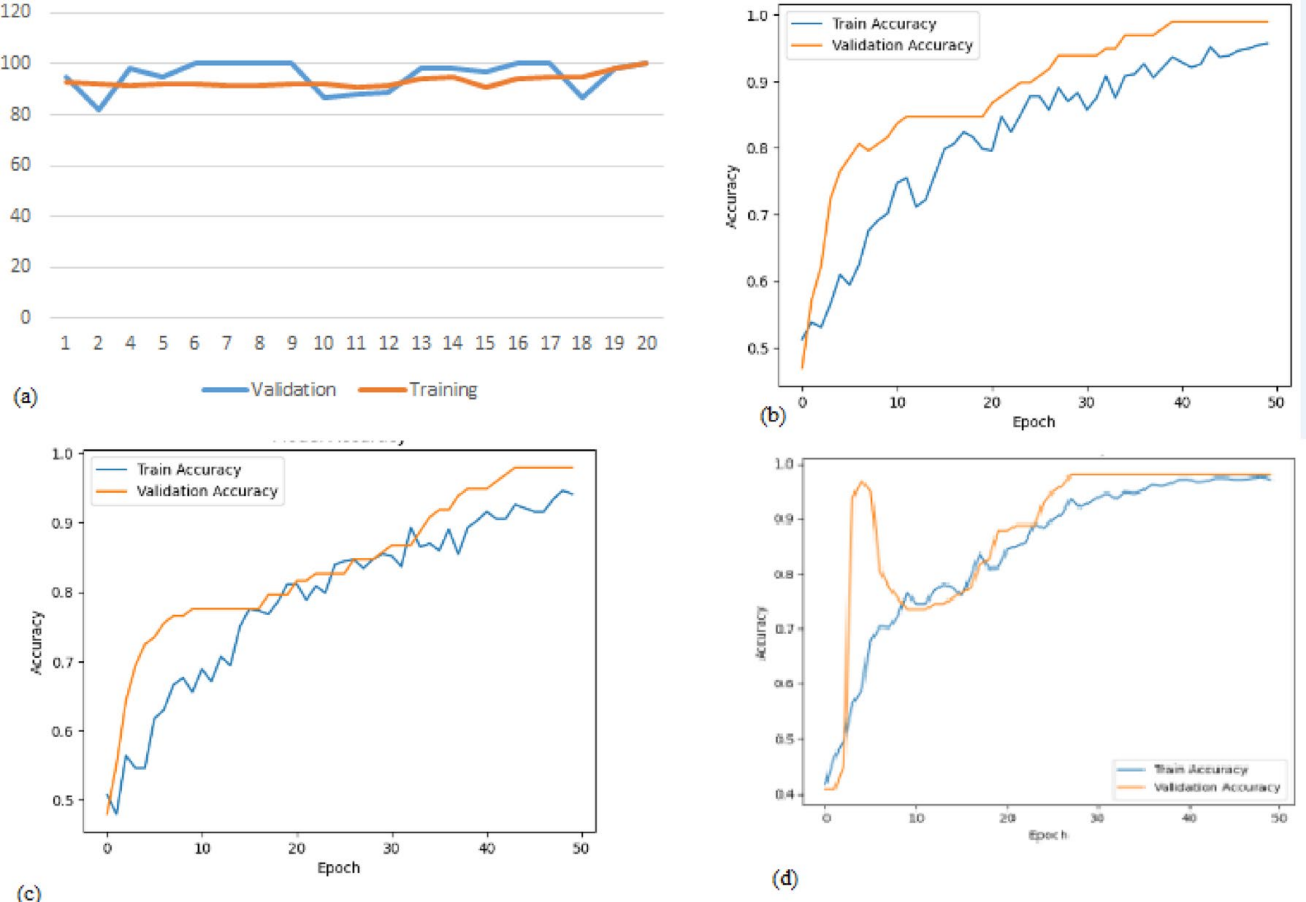
The validation and training accuracy for (**a**) Resnet, (**b**) VGG19, (**c**) VGG16, (**d**) Inception-V2.

Our proposed model was trained for 20 epochs with a learning rate set to 0.0001. The results demonstrate that the validation accuracy can reach up to 100%, marking a significant milestone in Lupus Erythematosus diagnostic accuracy, as illustrated in Fig. 11 due to converting ECG to audio data. When compared to all the diagnostic models reviewed in the “Related Works” section, our proposed model has demonstrated superior accuracy across the board, outperforming them in an efficient manner. This underscores the effectiveness and robustness of our model in achieving high diagnostic accuracy for Lupus Erythematosus 19. By training different models for the feature extraction, as shown in Fig. 12. We compared our models with efficient previous works and found that our model achieved the best performance, not only in accuracy but also other performance metrics, as shown in Table 4.

Table 4 presents a comprehensive comparison between our proposed model and recent state-of-the-art methods. While previous models like^3,5^ reported high accuracy (97.5% and 96.7%, respectively), our model surpasses all others with an accuracy of 99%, sensitivity of 99.2%, specificity of 97%, and F1-score of 98.1%. These results reflect not only high classification precision but also robust generalization across both Lupus and non-Lupus cases. Notably, although models^3,5^ achieved competitive sensitivity (96.7% and 96.2%) and F1-scores (94% and 95%), our model still outperformed them in every metric. This hybrid approach enables the capture of both morphological and spectral patterns, leading to superior diagnostic capability. Furthermore, the relatively high specificity (97%) reduces the risk of false positives, which is critical in clinical decision-making to prevent misdiagnosis.

To clarify the contribution of each subsystem, we conducted an ablation study comparing the performance of the individual ResNet-LSTM subsystem, the AST-only subsystem, and the full hybrid ensemble, as shown in Table 5. The results confirm that while both subsystems perform well independently, the hybrid ensemble consistently outperforms them across all key metrics demonstrating the complementary strengths of morphological and spectral feature learning.

**Table 4:** A comparison among our model and recent and efficient previous works.

References.	Acc	Sensitivity	Specifity	F1-Score
[1]	70%	-	73.2%	76%
[2]	81.4%	82.2%	79%	85%
[3]	97.5%	96.7%	94.1%	94%
[4]	93.1%	90.7%	-	89.6%
[5]	96.7%	96.2%	98.1%	95%
Proposed	99%	99.2%	97%	98.1%

9 $$\:Sen\:=\frac{TP}{TP+FN}$$

10 $$\:Spec\:=\frac{TN}{TN+FP}$$

11 $$\:Acc\:=\frac{TP+TN}{TP+FN+TN+FP}$$

**Table 5:** Results of the two sub-systems and hybrid system.

Model	Accuracy (%)	Sensitivity (%)	Specificity (%)	F1-score (%)
ResNet-LSTM only	96.3	96.5	94.2	95.3
AST only	97.1	97.8	95.1	96.4
Hybrid (proposed)	99.0	99.2	97.0	98.1

#### A creative comparison between the general diagnostic method and our proposed approach

**Fig. 13:**
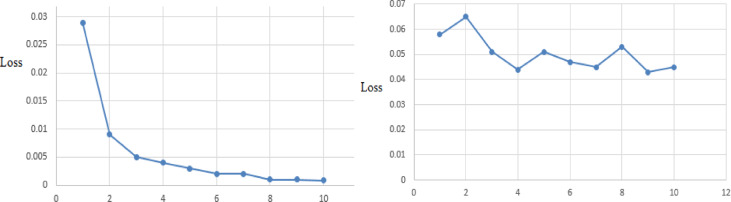
(**a**) loss of proposed model (**b**) loss of general approach.

In our innovative comparison between the general diagnostic method, for Lupus Erythematosus 19, and the one we propose, we differ from previous studies by developing a Lupus Erythematosus diagnostic model specifically for heart patients. This evaluation is based on the “validation-loss” metric, as depicted in Fig. 13. When compared to the general Lupus Erythematosus diagnostic model, which applies to individuals regardless of their pre-existing health conditions, we found that the validation loss exhibited inconsistency, showing fluctuations between upward and downward trends. Such instability indicates that the data has significant dispersion, implying a less efficient learning process. On the other hand, the validation-loss metric of our proposed model, designed exclusively for heart patients, demonstrates a steady decline, reaching a very small value of 0.0008, which is nearly zero. This suggests that our approach outperforms the general Lupus Erythematosus diagnostic model in terms of both learning efficiency and performance. The stable decrease in validation loss highlights the superior learning and enhanced performance of our model, specifically designed for heart patients in the context of Lupus Erythematosus diagnosis.

**Fig. 14:**
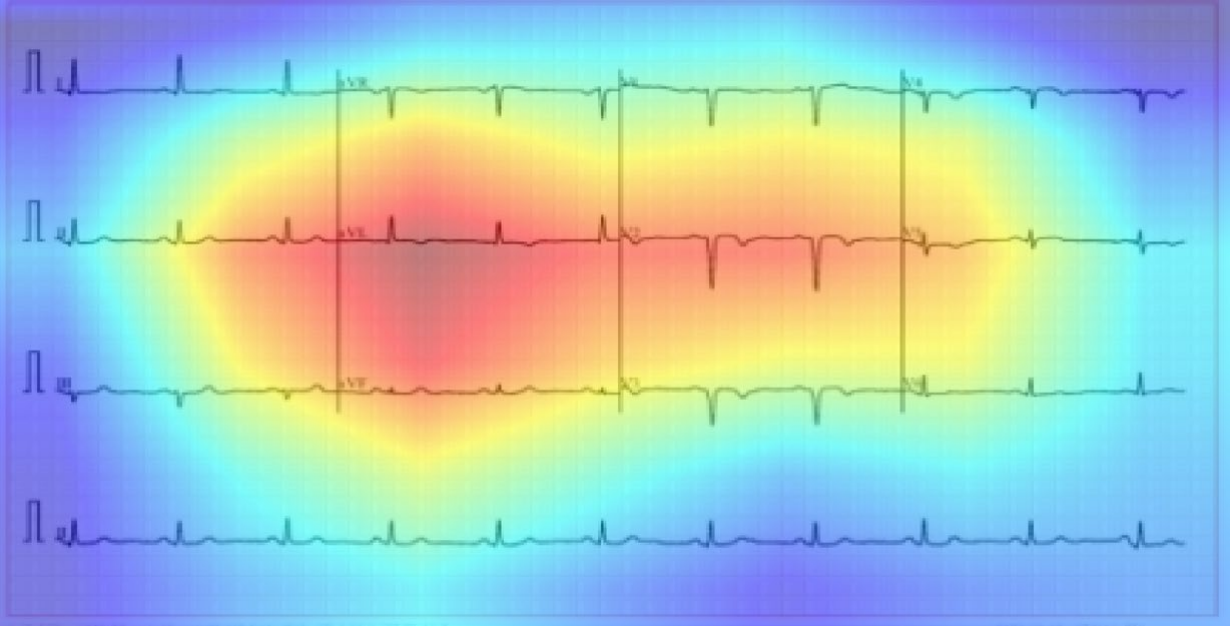
Heatmap of an ECG sample from a heart patient diagnosed with Lupus Erythematosus 19.

### Interpretation parameter

The interpretability of deep learning models is essential, as it provides clarity and reasoning for the decisions made by the model. Understanding the rationale behind specific predictions or classifications improves transparency and builds trust in its output. As illustrated in Fig. 14, our model concentrates primarily on the significant changes in heartbeats within the ECGs, disregarding other features. This highlights that our model gives priority to this particular characteristic when making decisions. The focus on a specific feature reflects the model’s learning patterns, allowing for a more straightforward interpretation of the factors driving its predictions. This focus on relevant features not only improves the interpretability of our model but also strengthens confidence in its decision-making abilities.

Our model’s focus on the QRS complex during classification was supported by Grad-CAM heatmap visualizations, which consistently highlighted this region in ECGs of LE-positive heart patients. This emphasis aligns with established clinical findings. Studies have reported that systemic inflammation and autoimmune responses in Lupus Erythematosus can lead to myocardial fibrosis and ventricular remodeling, manifesting as QRS prolongation or abnormal morphology in ECG readings^33^. In particular, left ventricular hypertrophy (LVH)—a known risk in LE patients—can significantly affect the QRS duration and amplitude. Our model’s attention to these regions is thus clinically explainable and reinforces its diagnostic relevance.

To quantitatively evaluate the relevance of Grad-CAM heatmaps, we conducted a controlled validation using 50 ECG samples. For each sample, bounding boxes were manually drawn around the most activated regions of the heatmap, as shown in Table 6. A domain cardiologist annotated expected zones of diagnostic importance (e.g., QRS, T-wave, ST-segment). We then computed: 1) Region Attention Accuracy (RAA): the percentage of samples where the model’s most activated region overlapped with the clinically expected zone. 2)Overlap with QRS Region (PO**)**: the proportion of model-activated area intersecting with the QRS complex, a key diagnostic marker in LE-related hypertrophy.

**Table 6:** Quantitatively analysis to heatmap outputs.

Metric	Mean (%)	Std. Dev. (%)
Region Attention Accuracy	88.4	±4.1
Overlap with QRS zone	91.2	±3.6

These results demonstrate that the model’s interpretability mechanism is not only visually explainable but also quantitatively aligned with clinical knowledge, validating its diagnostic reliability.

**Fig. 15:**
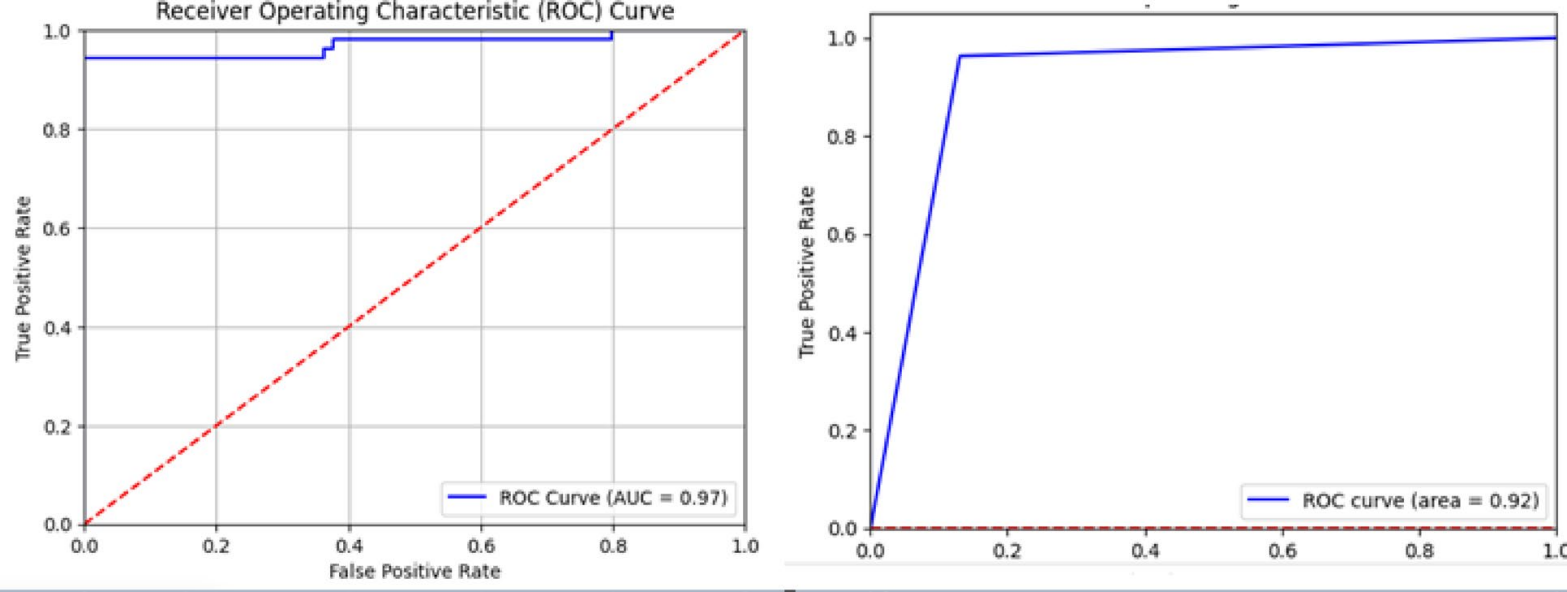
Auc for the two sub-systems.

### Receiver operating characteristics (ROC)

The ROC curve demonstrates the balance between sensitivity (true positive rate) and the false positive rate, providing insight into how effectively the model differentiates between positive and negative classes at various thresholds. In essence, the ROC curve offers a holistic view of the model’s discriminative ability, aiding in model selection and optimization processes. Our model has attained an ROC score of 92%, suggesting that it performs excellently in distinguishing between classes. Furthermore, our first subsystem has achieved an AUC of 97%, as shown in Fig. 15.

### Cross-Validation results

To evaluate model robustness and reduce the risk of overfitting, we conducted 5-fold stratified cross-validation across the entire dataset. Each fold preserved class distribution to ensure fair representation of LE-positive and normal ECGs. Table 7 reports the average and standard deviation across all five folds. These stable results across folds support the model’s generalizability and suggest that the high metrics reported are not the result of overfitting to a single data partition.

**Table 7:** Average and standard deviation for five folds.

Metric	Mean (%)	Std. Deviation (%)
Accuracy	98.85	± 0.52
Sensitivity	99.1	± 0.36
Specificity	96.8	± 0.44
F1-score	98.0	± 0.49

## Conclusion and future works

Through practical demonstrations, we observed that using a general model for diagnosing Lupus Erythematosus ineffective due to data dispersion, which negatively impacts performance. As a solution, this study introduces a deep learning-based diagnostic model for Lupus Erythematosus specifically tailored for heart patients. Our proposed model shows highly efficient results. To align the model with the only available ECG dataset for Lupus Erythematosus patients, we collaborated with a cardiology expert. We refined this dataset by categorizing it into two distinct classes: ECGs of heart patients with confirmed Lupus Erythematosus cases and ECGs of heart patients without Lupus Erythematosus 19. This careful dataset refinement enhances the model’s relevance and contributes to its strong performance in diagnosing Lupus Erythematosus among heart patients.

Our study also presents a novel ECG-to-audio transformation framework that leverages the capabilities of advanced audio-based deep learning models for the classification of cardiac conditions, including those associated with Lupus Erythematosus. By converting ECG signals from image format into normalized audio waveforms and subsequently into Mel-spectrograms, this approach enables the extraction of rich time-frequency features that are often overlooked in conventional time-domain or image-based analyses. The application of the Audio Spectrogram Transformer (AST), fine-tuned for the classification of spectrograms, has demonstrated superior performance in identifying subtle morphological variations in ECG patterns, achieving high classification accuracy and interpretability as a novel work. Our results outperform previous models, demonstrating superior efficiency. Furthermore, we validate the effectiveness of our model using an explainable deep learning approach through a heatmap algorithm, where the model emphasizes significant changes in heartbeats in the ECGs while disregarding other features. This shows that our model prioritizes this specific feature in its decision-making process. Our findings indicate that Lupus Erythematosus may lead to hypertrophy in the heart muscle, especially in the ventricles, as our algorithm highlights the elevated QRS region in the ECG images from the test dataset. This outcome underscores the connection between Lupus Erythematosus and its impact on the heart.

The clinical deployment of AI-based diagnostic systems must be guided by ethical considerations, particularly when outcomes may directly influence patient care. In our model, a false positive (misclassifying a healthy patient as LE-positive) could lead to unnecessary anxiety, follow-up tests, or treatment. Conversely, a false negative may delay critical intervention for an LE patient with cardiac risks, potentially worsening prognosis. While our system demonstrates strong sensitivity (99.2%) and specificity (97%), no model is infallible. Therefore, we emphasize that the tool is intended to support—not replace—clinical judgment. Ethical safeguards, including clinician-in-the-loop systems and informed consent during future trials, are essential to minimize harm. Furthermore, interpretability mechanisms (e.g., heatmaps) aid transparency, allowing clinicians to understand and trust the system’s predictions.

While the proposed hybrid diagnostic model demonstrates excellent performance, several limitations must be acknowledged. First, the dataset size remains relatively limited, particularly for confirmed Lupus Erythematosus (LE) cases with cardiac comorbidities. This can affect the model’s generalizability, especially across diverse demographic or clinical subgroups. Second, the model has not yet been tested in real-world clinical settings or validated using external datasets. Although we collaborated with a cardiology expert to curate the ECG categories, true deployment would require prospective clinical trials. Finally, the dataset lacks comprehensive metadata such as ethnicity or medication status, which could further influence ECG features. Future work will focus on collecting larger, demographically diverse datasets, validating the model in hospital settings, and exploring lightweight architectures for deployment in mobile health systems.

## Data Availability

The dataset used in this study is available upon reasonable request. Interested researchers may contact Ahmed Hassan at ahmedhassancs22@yahoo.com.
